# Oncology Guidelines Usage in a Low- and Middle-Income Country

**DOI:** 10.1200/JGO.17.00136

**Published:** 2018-04-11

**Authors:** Nofisat Ismaila, Omolola Salako, Jimoh Mutiu, Oladeji Adebayo

**Affiliations:** **Nofisat Ismaila**, American Society of Clinical Oncology, Alexandria VA; **Omolola Salako**, Lagos University Teaching Hospital, Lagos; **Jimoh Mutiu**, University College Hospital; and **Oladeji Adebayo,** University of Ibadan, Ibadan, Nigeria

## Abstract

**Purpose:**

There is a paucity of data about current usage of oncology guidelines in low- and middle-income countries (LMICs), specifically in terms of the availability and quality of those guidelines. Our objective was to determine usage of oncology guidelines and the barriers and facilitators to their usage among radiation oncologists in LMICs.

**Methods:**

An online cross-sectional survey was conducted among practicing radiation oncologists in Nigeria via e-mail and the social media database of the Association of Radiation and Clinical Oncologists of Nigeria. In addition, paper questionnaires were administered at regional clinical meetings.

**Results:**

The survey response rate was 53.4% in a sample of 101 radiation oncologists from the database. Sixty-nine percent of respondents were consultants and 30% were residents. Approximately 43% had < 5 years’ experience. All of the respondents were involved in administering chemotherapy during the treatment of patients with cancer, whereas approximately half were involved in diagnosing cancer. Ninety-three percent reported using guidelines in treating patients, the top two guidelines being those from the National Comprehensive Cancer Network (90%) and the American Society of Clinical Oncology (50%). The two major barriers to guideline usage were that facilities were inadequate for proper guideline implementation and that the information in guidelines were too complex to understand. Potential facilitators included providing adequate facilities, developing local guidelines, and increasing awareness of guideline usage.

**Conclusion:**

Our study shows that clinicians involved in the treatment of patients with cancer in LMICs are aware of cancer treatment guidelines. However, implementation of these guidelines hinders their usage because the facilities are inadequate, guidelines are not applicable to the local setting, and the information in the guidelines is too complex.

## INTRODUCTION

Clinical decisions that determine outcomes, quality of life, and survival rate are made on a daily basis in low- and middle-income countries (LMICs). Thus, there is a need to streamline the decision-making process with a set of instructions that offers guidance on how to manage a particular clinical scenario. This can be achieved by using clinical treatment guidelines also known as practicing evidence-based medicine. Treatment guidelines are used across all fields of medicine to ensure quality care, standardized care, and cost control. The Institute of Medicine defines clinical practice guidelines as “. . . statements that include recommendations intended to optimize patient care that are informed by a systematic review of evidence and an assessment of the benefits and harms of alternative care options.”^1^ These guidelines have been established to improve patient care.^1^ The Guidelines International Network currently contains more than 6,400 guidelines from 85 countries.^2^ Most of these guidelines are created by reputable guideline development organizations that are based in developed countries. These treatment guidelines are used globally by health care providers; however, the use of guidelines by health care providers varies according to the region, available facilities, patient demographics, health care funding, disease presentation, health care personnel, and expertise in providing health care.

Cancer is the leading cause of death worldwide, accounting for approximately 8.2 million deaths in 2012.^3^ In the developed world, cancer is regarded as a preventable and often curable disease. Unfortunately, in many LMICs, the mortality and morbidity rates are unacceptably high, making cancer a death sentence.^4^ There is a growing cancer crisis in the developing world.^3,5^ Annually, more than 60% of the world’s total new cases occur in developing countries, specifically in parts of Africa, Asia, Central America, and South America.^5^ It is estimated that nearly 15 million people will have a high probability of being diagnosed with cancer in the year 2015, with the majority occurring in developing countries.^5,6^ This rapid increase has been associated with increasing life expectancy, changing lifestyles, and unhygienic living conditions.^3,6^ For example, Nigeria, which is categorized as an LMIC, is experiencing an increasing incidence of cancer cases.^7^ The most common cancers in Nigeria are cancer of the prostate and liver in men and cancer of the breast and cervix in women.^7^

Across Nigeria, there is currently a shortage of cancer specialists, such as surgeons, radiation oncologists, and medical oncologists—the physicians who recommend, prescribe, and administer chemotherapy—with less than five medical oncologists and approximately 100 radiation oncologists (consultants and residents in training) in the country.^8^ The majority of radiation oncologists are practicing in radiotherapy centers and are trained in the administration of chemotherapy.^8^ These radiation oncologists spend a significant portion of their training and practice prescribing and administering chemotherapy and managing adverse effects.

In addition, patients are treated on the basis of access to resources at the institution that provides their care. Resources include the availability of cancer specialists with various levels of expertise in administering chemotherapy, availability of anticancer agents, the cost of chemotherapy, and the availability of supportive care, which may lead to wide disparity in how patients are treated. An overview of the disparities in availability, usage, quality, and barriers to implementation of cancer treatment guidelines in Nigeria has not been studied. Hence, this national, cross-sectional survey was conducted to provide an overview of the use of cancer treatment guidelines among radiation oncologists in Nigeria.

The primary objective of this study was to measure the level of use of cancer treatment guidelines among health care providers in Nigeria who are involved in the treatment of patients with cancer. The secondary objectives were to identify potential barriers to the use of cancer treatment guidelines, and potential facilitators of their use.

## METHODS

This study was implemented in two phases. In the first phase, reported here, we focused on radiation oncologists. The second phase would be an expansion study to include other health care providers involved in managing patients with cancer in Nigeria.

### Study Design

The study used a cross-sectional, prospective survey conducted between December 2016 and February 2017. The survey examined the use of cancer treatment guidelines to identify potential barriers to and facilitators for their use.

### Sample Population

The Association of Radiation and Clinical Oncologists of Nigeria (ARCON) database was used to capture the population of radiation oncologists in Nigeria. The ARCON database has approximately 100 radiation oncologists spread across 10 states in Nigeria, which ensured that different parts of the country would be represented in the collected data.

### Survey Tool

We used a self-reported questionnaire that consisted of three main sections and required approximately 5 to 7 minutes to complete. The first section included data on guideline usage and demographic characteristics such as the participant’s name, institutional affiliation and location, number of years in practice, area of specialization, guideline usage, and specific guidelines used. The second section consisted of a self-reported 19-item scale, which was adapted from the validated barriers to research utilization scale (BARRIERS) developed by Funk et al^9^ in 1991. Respondents were asked to rate each item on what they perceived to be barriers to using cancer treatment guidelines in their practices on a 5-point Likert scale from 1 (strongly disagree) to 5 (strongly agree). The third section, in an open comment format, was structured to elicit responses on the top three potential facilitators to usage of cancer treatment guidelines.

### Ethical Consideration

Ethical approval was received from the ethics committee of the Lagos University Teaching Hospital. An introductory letter with full information about the study was included with the invitations to complete the online questionnaire sent via e-mail, or via postage for paper copies. The completion of the questionnaire, online or in person for paper versions, was taken to imply consent to participate in the study. All data received from the questionnaire were kept confidential and were accessed only by the researchers. All participants in the study were assured that their participation was voluntary.

### Data Collection

Survey data were collected electronically using the Survey Monkey platform and also by in-person administration of paper surveys at meetings. An e-mail to request the completion of the questionnaire was sent to those in the ARCON e-mail database. The questionnaire was sent as a Web link (via Survey Monkey) and as a Word (Microsoft, Redmond, WA) attachment. In addition, the questionnaire was also distributed at two oncology conferences that targeted radiation oncologists in Nigeria. Reminders were sent via e-mail and by telephone on weeks 1 and 3 after the first e-mail invitation.

### Data Analysis

All data collected electronically and on paper were entered manually into the Survey Monkey online database. Individual and summary data were then downloaded into an Excel file (Microsoft, Redmond, WA) and were numerically coded for quantitative analysis. Descriptive statistics such as frequency mean and standard deviation were used to describe the basic demographic characteristics of the respondents and to summarize the modified BARRIERS scores. Data collected on potential facilitators was coded and analyzed.

## RESULTS

### Demographic Characteristics of Respondents

A total of 54 radiation oncologists completed the survey questionnaires, yielding a response rate of 53.4%. A summary of the demographic characteristics of all respondents is listed in Table 1. A majority of the respondents (68.5%) were consultants, and residents made up approximately 30%. The percentage of respondents who had < 5 years of experience or between 5 and 10 years of experience was 42.6% and 40.7%, respectively. Only 17% had more than 10 years of experience managing patients with cancer.

**Table 1:**
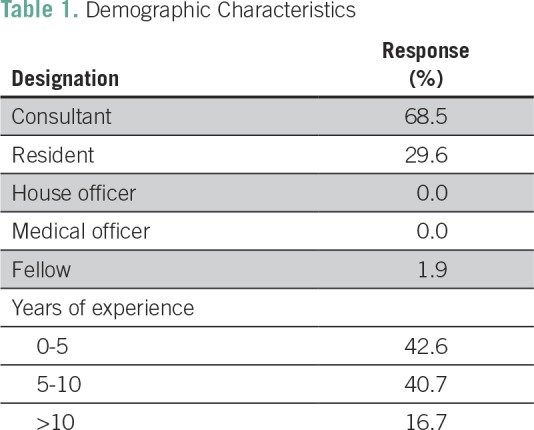
Demographic Characteristics

### Experience Treating Patients With Cancer

**Fig 1:**
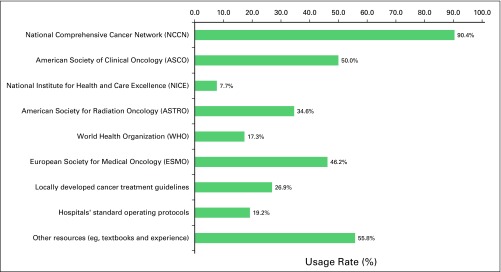
Guidelines used in practice.

Almost all of the radiation oncologists reported being involved in the radiotherapy (98%) and chemotherapy (100%) administration phase of treating patients, followed by involvement in supportive care (92.6%), diagnosis (61.1%), and screening (51.8%). When asked if they used cancer treatment guidelines or any specific standard treatment protocol in treating patients in their respective practices, 92.6% said, “Yes” and 7.4% said, “No.” Figure 1 shows the details of specific guidelines that were being used. The top three guidelines were from the National Comprehensive Cancer Network (NCCN; 90%), American Society of Clinical Oncology (ASCO; 50%), and the European Society of Medical Oncology (ESMO; 46%).

### Perceived Barriers to Guideline Usage

The top three major barriers identified were inadequate facilities for properly implementing guidelines, guideline information that was too complex or overwhelming, and guidelines that were not applicable to the setting with average ratings of 2.88, 2.57, and 2.47, respectively. The barriers that ranked lowest were guidelines that were not readily available, physicians being unaware of the guidelines, and the guidelines not being relevant to the physician’s practice. The 19 barriers discussed in the questionnaire along with their ratings are listed in Table 2.

**Table 2:**
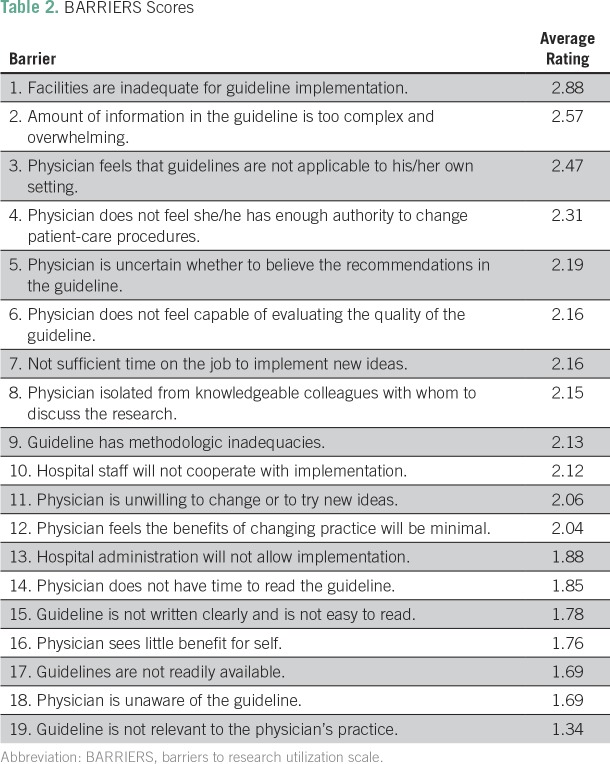
BARRIERS Scores

### Potential Facilitators of Guideline Usage

**Fig 2:**
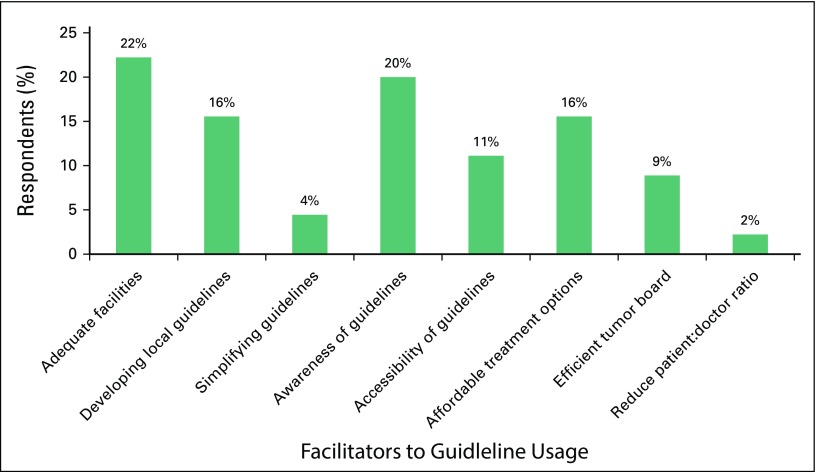
Identified facilitators to guideline usage.

Data for this section were collected in an open comment format. Eight themes emerged from the qualitative analysis and were coded accordingly. The frequency of response for each theme is shown in Figure 2. The top three facilitators were providing adequate facilities (22%), increasing awareness of the use of guidelines (20%), and developing local guidelines (16%). Reducing the patient:physician ratio and simplifying guidelines had the least support.

## DISCUSSION

To our knowledge, this was the first ever study conducted among radiation oncologists in Nigeria to examine guideline usage and possible barriers to and facilitators of their usage. One of the limitations of this study was the average response rate of the survey. The response rate was partly a result of the fact that the survey was mostly available online, and most physicians were either too busy to complete the survey or had limited access to the Internet. This average response rate may also be a result of clinicians not being familiar with the guidelines; thus, they were unable to comment on guideline usage. We made an attempt to improve coverage and response by administering some hard copies of the questionnaires at two conferences; however, this covered only a small percentage of the study population. A similar study was conducted by Kerr et al^10^ in 11 LMICs, although they focused mainly on guidelines for breast and lung cancer. They reported having 139 survey respondents, but they have not yet published the full details of the outcome of their study.^10^

Regarding the usage rate for cancer treatment guidelines, this study showed that almost all of the radiation oncologists sampled used some form of guideline document when treating their patients. A majority used guidelines developed by NCCN, followed by ASCO and ESMO. However, approximately half the respondents also reported that they obtained guidance from other non-guideline resources (eg, textbooks, manuals, or hospital protocols). This study reported findings similar to those of Kerr et al,^10^ who reported that 92% of their respondents used NCCN, 55% used ASCO, 55% used ESMO, and 40% used national guidelines. Despite the high rate of usage of these guidelines, several barriers were reported regarding their proper usage or implementation, the largest of which was inadequate facilities (eg, lack of radiation machines, infusion centers, oncology clinics, and a stable electric power supply). Because the current guidelines were created in developed countries where clinical data and resources are readily available, implementing some of the recommendations was a major challenge in a setting that has suboptimal infrastructure and resources. However, several of the organizations that develop guidelines have started working toward providing guidance for clinicians who work in a limited-resource setting. For example, international organizations such as the WHO, International Agency for Research on Cancer, ASCO, NCCN, and Breast Health Global Initiative (BHGI) have ongoing programs that provide cancer treatment guidelines based on resource availability. For example, BHGI and NCCN have developed several resource-stratified guidelines, and in 2016, ASCO issued its first resource-stratified treatment guidelines for managing invasive cervical cancer.^11^

Implementing international guidelines in the African setting is challenging, and often, it is not feasible to use the guidelines for making clinical decisions. In Nigeria, several cancer specialists and local organizations continue to explore the development of locally relevant guidelines or the modification of international guidelines to suit the limited health care resource setting and diverse pattern of patient presentation. To address this issue, in 2006, the Federal Ministry of Health created a national chemotherapy committee that developed a chemotherapy guideline as part of the National Cancer Control Plan. However, after the guideline was disseminated, it was not widely implemented in the clinical setting. Recently, the first edition of a breast cancer pocket guideline was developed by a charity in collaboration with the Federal Ministery of Health. It was launched in March 2017 and is currently being disseminated.^12^

The number one barrier reported in the survey was also the number one facilitator. Hence, if resources were made available to improve facilities and provide more efficient infrastructures, implementation of these guidelines would be smoother and less challenging.

In summary, despite the inherent bias that comes with a self-reporting survey, the data generated from this study provide evidence that can be used to guide the development of future locally relevant cancer treatment guidelines that factor in existing resources and constraints and address how they can be optimally applied to improve cancer treatment outcomes.
